# Effect of tartary buckwheat, rutin, and quercetin on lipid metabolism in rats during high dietary fat intake

**DOI:** 10.1002/fsn3.1291

**Published:** 2019-11-27

**Authors:** Lianxin Peng, Qu Zhang, Yanhong Zhang, Zhendong Yao, Panpan Song, Lijuan Wei, Gang Zhao, Zhuyun Yan

**Affiliations:** ^1^ Key Laboratory of Coarse Cereal Processing of Ministry of Agriculture and Rural Affairs Chengdu University Chengdu China; ^2^ Pharmacy College Chengdu University of Traditional Chinese Medicine Chengdu China

**Keywords:** gut microbiota, lipid metabolism, quercetin, rutin, tartary buckwheat

## Abstract

Tartary buckwheat is rich in flavonoids. However, the health‐promoting effect of these flavonoids has not been adequately studied. In the present study, we investigated the impact of rutin, quercetin, and Tartary buckwheat on the lipid metabolism of rats on a high‐fat diet. Quercetin could significantly reduce body weight, serum triacylglycerol, low‐density lipoprotein cholesterol, TNF‐α, insulin, and ameliorate glucose tolerance. It was surprising that Tartary buckwheat significantly increased the weight of the rats. Rutin, quercetin, and Tartary buckwheat tended to decreased fat deposition in the liver of rats but have little effect on short‐chain fatty acid production. The changes in the structure and diversity of the microbiota were found to be modulated by these diets. It was concluded that quercetin could attenuate high‐fat diet‐induced obesity, rutin, quercetin, and Tartary buckwheat can shape the specific structure of gut microbiota. Mechanism of Tartary buckwheat on lipid metabolism needs further systematic research.

## Graphical abstract

Tartary buckwheat is rich in flavonoids, and however, the health‐promoting effect of these flavonoids has not been adequately studied. This study reports the impact of rutin, quercetin, and Tartary buckwheat on the lipid metabolism and gut microbiota of rats on a high fat diet.

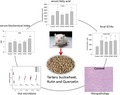

## 1 INTRODUCTION

Obesity is often accompanied by a series of health problems, such as insulin resistance, type 2 diabetes, fatty liver disease, atherosclerosis, and degenerative diseases such as dementia, respiratory diseases, and various types of cancer (Engin, 2017; Kawasato et al., 2017). Globally, more than 1.9 billion adults are overweight and more than 600 million obese (WHO, 2015). Therefore, obesity and the various metabolic diseases caused by obesity have become a threat to human health and the quality of life in the 21st century. Obesity is caused mainly by energy imbalance, which appears as glucolipid metabolism disorders. Reports have shown the great potential of a reasonable diet on the prevention and treatment of obesity although what constitutes a reasonable diet is still controversial (Berberich & Hegele, 2018). More research has focused on the effect of food nutrients such as dietary fiber, resistant starch, polysaccharide, and polyphenols on obesity and its associated diseases, with findings indicating that this type of nutritional intake can improve metabolism (Farhat, Drummond, & Al‐Dujaili, 2017; Kasprzak, Wojtunik‐Kulesza, Oniszczuk, Kuboń, & Oniszczuk, 2018; Zhang et al., 2015).

Gut microbiota (GM) have been reported to be involved in obesity, glucolipid metabolism, and cardiovascular disease (van de Wouw, Schellekens, Dinan, & Cryan, 2017). Intestinal flora in the host plays an important role in energy metabolism and balance through adjusting the molecular signals released. The contents of signaling molecules such as ghrelin, leptin, and glucagon peptide (glp‐1) have shown excess or insufficiency on the body's energy (van de Wouw et al., 2017). Dietary or drug intervention to regulate the structure of intestinal microbes has been demonstrated to have a positive effect on both humans and animals (Li, Watanabe, & Kimura, 2017; Sonnenburg et al., 2016).The metabolites of gut microbes with food, such as short‐chain fatty acids (SCFAs), bile acid, GABA, dopamine, and norepinephrine, play an important role in changing dietary habits and host metabolism, and in maintaining weight and energy balance by affecting signaling molecules through gut‐brain axis adjustment (van de Wouw et al., 2017). SCFAs, produced by GM degrading nondigestible carbohydrates, have a potentially inhibitory effect on inflammation and can be an important regulatory factor in human metabolism. However, the relationship between GM, SCFAs, and glucolipid metabolism is still unclear, so more research is required.

Epidemiological data have indicated that increasing the intake of grains, vegetables, and fruit can significantly reduce the risk of metabolic syndromes (MS) such as obesity (Miller et al., 2017). Flavonoids exist widely in fruits, vegetables, and whole grains, and exhibit a variety of physiological activities such as regulating gut microbiota, and antioxidative and anti‐inflammatory effects (D'Andrea, 2015). Previous reports have indicated the modulatory capacity of flavonoids on the gut microbial community (Gil‐Cardoso et al., 2016). Recent studies have indicated that quercetin and rutin can modify the gut microbial balance, and alleviate obesity and liver disease (D'Andrea, 2015; Panchal, Hemant, Arumugam, & Brown, 2011; Porras et al., 2016). Thus, the use of food rich in flavonoids, such as quercetin, to metabolize lipids might be a potential strategy for modulating the composition of intestinal bacteria. Tartary buckwheat, belonging to the genus *Fagopyrum* of the Polygonaceae family, has been reported to help control blood glucose, blood pressure, and blood lipid levels (Zhu, 2016). Tartary buckwheat is rich in flavonoids, mainly rutin (Zhu, 2016), and has therefore been attracting more research attention. However, it needs to be clarified whether dietary rutin or quercetin in buckwheat can ameliorate lipid metabolism, because the starch and protein in buckwheat flour may also have beneficial effects on lipid metabolism. Meanwhile, our previous study indicated that the nutritive active components of Tartary buckwheat were related to different parts and processing methods (Peng et al., 2017, 2015). Thus, the real effect of these flavonoids present in buckwheat needs further studies to promote the development and utilization of Tartary buckwheat.

The present study aims to investigate the potential benefits of an experimental treatment with quercetin, rutin, and Tartary buckwheat on high‐fat diet fed rats on regulating lipid metabolism, restoring host microbial balance, and regulating the SCFAs signaling mechanisms for MS. The results will improve the understanding of the functional properties of buckwheat, and also the design of therapeutic approaches to manipulate gut microbiota for treating MS or malnutrition.

## 2 MATERIALS AND METHODS

### 2.1 Materials

Rutin and quercetin were purchased from Wuhan Yuancheng Gongchuang Technology Co. Ltd, Tartary buckwheat (*Fagopyrum tataricum* cv. Chuanqiao No. 1) (sieved through 80–120 mesh screens) from Huantai industrial co. Ltd., fatty acid standards from Sigma‐Aldrich Co., and acetonitrile (HPLC‐grade) from Fisher Scientific Co. All other chemicals and solvents used in the study were of analytical grade.

### 2.2 Animals and treatments

All experiments on animals were performed in strict compliance with the guides for the Care and Use of Laboratory Animals published by US National Institutes of Health and was approved by the Use Committee of Chengdu University. Seven‐week‐old male Sprague‐Dawley (*SD*) rats (220 g ± 20 g, Permission Number: SCXK (Chuan) 2018–19) were fed with a standard diet to adapt to the environment then divided into five groups (ten rats per group): control (AIN 93‐G), HFD (high‐fat diet), HFDQ (HFD containing 0.1% quercetin), HFDR (HFD containing 0.2% rutin), and HFDB (HFD + Tartary buckwheat) (Table 1). Rats were fed the diets and water ad libitum, housed in standard polypropylene cages (five rats/cage), and maintained under a controlled room temperature (24 ± 2°C) and relative humidity (55 ± 5%) with a 12/12‐hr light/dark cycle. The body weights were monitored weekly, and food intake was monitored daily. After 12 weeks, the rats were euthanized, and then, serum, liver, feces, and adipose tissue samples were collected and weighed. The right posterior lobe of the liver was fixed in 10% formalin. All experimental protocols were approved by the Animal Experimentation Ethics Committee of Chengdu University for the care and use of laboratory animals.

**Table 1:** Composition of diets in animal experiment

Composition	Control	HFD	HFDR	HFDQ	HFDB^a^
Casein	20.0%	20.0%	20.0%	20.0%	20.0%
Dextrinized cornstarch	13.2%	0	0	0	0
Sucrose	10.0%	10.0%	10.0%	10.0%	10.0%
Corn starch	39.7486%	0	0	0	0
Steamed wheat flour	0	32.9492%	32.7492%	32.8492%	
Steamed wheat and buckwheat flour (7:3)^b^	0	0	0	0	32.9492%
Alphacel, non‐nutritive	5.0%	5.0%	5.0%	5.0%	5.0%
Soybean oil	7%	7%	7%	7%	7%
AIN−93G mineral mix	3.5%	3.5%	3.5%	3.5%	3.5%
L‐Cystine	0.3%	0.3%	0.3%	0.3%	0.3%
AIN−93‐VX vitamin mix	1.0%	1.0%	1.0%	1.0%	1.0%
Choline bitartrate	0.25%	0.25%	0.25%	0.25%	0.25%
tert‐Butylhydroquinone	0.0014%	0.0014%	0.0014%	0.0014%	0.0014%
Lard	0%	20%	20%	20%	20%
Rutin	0	0	0.2%	0	0
Quercetin	0	0	0	0.1%	0

a Finally contained 0.27% rutin and 0.01% quercetin, using HPLC method.

b Steamed for 20 min at 100°C.

### 2.3 Biochemical analysis

The serum samples were assayed for triacylglycerol (TG), total cholesterol (TC), high‐density lipoprotein cholesterol (HDL‐C), low‐density lipoprotein cholesterol (LDL‐C), creatinine, and urea, and cystatin C levels were measured using assay kits (Changchun Huili Biotech Co., Ltd.). The fasting insulin, IL‐6, and TNF‐α levels were determined using commercial rat ELISA kits (ExCell Biology Inc., Taicang, China) according to the manufacturer's instructions. Oral glucose tolerance tests were performed after 11 weeks of feeding as follows: all the rats were fasted for 10 hr followed by an oral glucose load (2 g/kg of body weight); blood samples were collected from the tail vein, and glucose levels were determined using a Glucometer (Accu‐chek^®^ Performa, Roche Diagnostics) at 0, 30, 60, and 120 min after glucose administration.

### 2.4 Histopathology

Liver pathology was evaluated via hematoxylin and eosin (HE) staining. The livers were fixed in 10% neutral formalin, dehydrated in ethanol, and, then, embedded in paraffin. Tissue sections were stained with HE and observed under microscope (Olympus‐CX31). The degree of steatosis of the liver cells was observed under a microscope. A hepatic steatosis score (0–4) was given by a pathologist unaware of the diet treatment groups of the rats: 0 (steatosis < 5%), 1 (< 15% steatosis), 2 (< 30% steatosis), 3 (steatosis < 50%), or 4 (steatosis ≥ 50%).

### 2.5 Identification and quantification of fatty acids

The fatty acids were extracted and quantified as described by Wang, Li, Muhammad, and Zhang (2016) with slight modification. One hundred µl of a 10 mg/ml internal standard (Tridecane acid) and 2 ml petroleum ether were added to 1‐mL serum samples and, then, extracted using vortex oscillation for 3 min. After centrifugation at 10,000 g, the supernatant was collected. Extraction was repeated three times and the supernatants were combined, then dried using N_2_. After adding 3 ml 0.5 mol/L NaOH‐methanol, the mixture was shaken, heated at 60°C for 6 min, and then, 3 ml 5% boron trifluoride‐methanol was added and held at 60°C for 7 min. After cooling down to room temperature, 2 ml saturated NaCl and 1 ml n‐hexane were added, vortexed, left for 30 min, and then, the supernatant was collected. FFA were identified and quantified using a gas chromatograph coupled with a flame ionization detector (Agilent 7890A, Agilent Technologies Inc.). The samples were separated using a SPTM‐2560 capillary column (100 m × 0.25 mm × 0.2 μm; Supelco). The split ratio was set at 50:1, the injection volume was 1 μl, the injector temperature was 240°C, and the detector temperature was 250°C. The temperature program was as follows: initial temperature of 40°C, raised at 3°C/min to 220°C, raised at 1°C/min to 230°C, and then held there for 23 min. The flow rate of nitrogen was 1.0 ml/min.

### 2.6 Identification and quantification of cecal short‐chain fatty acids (SCFAs)

One hundred μl 15% H_3_PO_4_, 50 μg/ml internal standard (isocaproic acid), and 400 μl diethyl ether were added to 100‐mg cecal samples, homogenized for 1 min. After centrifugation for 10 min at 12,000 g and 4°C, the supernatant was collected. The SCFAs were identified and quantified on a gas chromatograph connected to a mass spectrometer (GC‐MS, 6890N/5975B, Agilent Technologies Inc.). The samples were separated using HP‐INNOWAX capillary column (30 m × 0.25 mm I.D., Agilent J&W Scientific). The split ratio was set at 10:1, the injection volume was 1 μL, the injector temperature 250°C, the ion source temperature 230°C, and the transmission line temperature 250°C. The temperature program was as follows: initial temperature of 90°C, raised at 10°C/min to 120°C, raised at 5°C/min to 150°C, and raised at 25°C/min to 250°C then held there for 2 min. The flow rate of nitrogen was 1.0 ml/min. Mass spectrometry was performed using the full‐scan and SIM scan methods. A linear regression equation (*R* ^2^ ≥ 0.99) was used to calculate the concentration of each SCFA from the standard curves obtained using seven different concentrations.

### 2.7 Cecal DNA extraction and sequencing

DNA was extracted from the cecal contents by a bead‐beating method using the ZR Fecal DNA MiniPrep Kit (Zymo Research Corp.) according to the manufacturer's instructions. DNA was detected by 0.8% agarose gel electrophoresis. The extracted DNA was used as the template to amplify the V3‐V4 region of the 16S rDNA gene using the 16S V3 314F forward primer (5’‐CCTAYGGGRBGCASCAG‐3’) and V4 806R reverse primer (5’‐GGACTACHVGGGTWTCTAAT‐3’). The PCR analysis was conducted on a thermocycler PCR system (GeneAmp PCR system 9,700; Applied Biosystems). The PCR conditions used were as follows: 1 min at 94°C, followed by 30 cycles of 20 s at 94°C, 30 s at 54°C, and 30 s at 72°C, with a final extension at 72°C for 5 min. Each reaction mixture (25 μl) contained 10 ng of genomic DNA, 1.0 µM of amplicon PCR forward primer, 1.0 µM of amplicon PCR reverse primer, 1x PCR buffer, 1.5 mM MgCl_2_, 0.4 µM dNTPs (deoxynucleotide), and 0.5 U KOD‐Plus‐Neo enzymes (Toyobo).

The products from different samples were sequenced pair‐end on the MiSeq Illumina Sequencing Platform with the sequencing strategy PE300 (MiSeq Reagent kit). All high‐quality sequencing reads were clustered using USEARCH (version 7.1, http://www.drive5.com) with 97% similarity, and operational taxonomic units (OTU) were obtained with a certain threshold. Each OTU was selected as the representative sequence for taxonomical assignment using the UCLUST algorithm. The number of sequences per sample was corrected for differences in sequencing depth between samples by rarefication, that is, the same number of reads was randomly subsampled in each sample. Then, the absolute number of sequences of each OTU in each sample was converted to relative abundance to reduce the effect of differences in sequence reads. The representative sequences, together with the abundance data, were used for taxon‐based analysis.

### 2.8 Statistical analysis

The data were expressed as mean ± *SD* and evaluated with one‐way analysis of variance (ANOVA) and Student's *t* test. *p* < .05 was considered to be significant for a difference between mean values. Alpha diversity and beta diversity were analyzed using a procedure in R. Principal coordinate analysis were using Bray–Curtis distances. All statistical analyses were performed using IBM SPSS Statistics (version 22.0, IBM Corp.).

## 3 RESULTS AND DISCUSSION

### 3.1 Serum biochemicals

**Figure 1:**
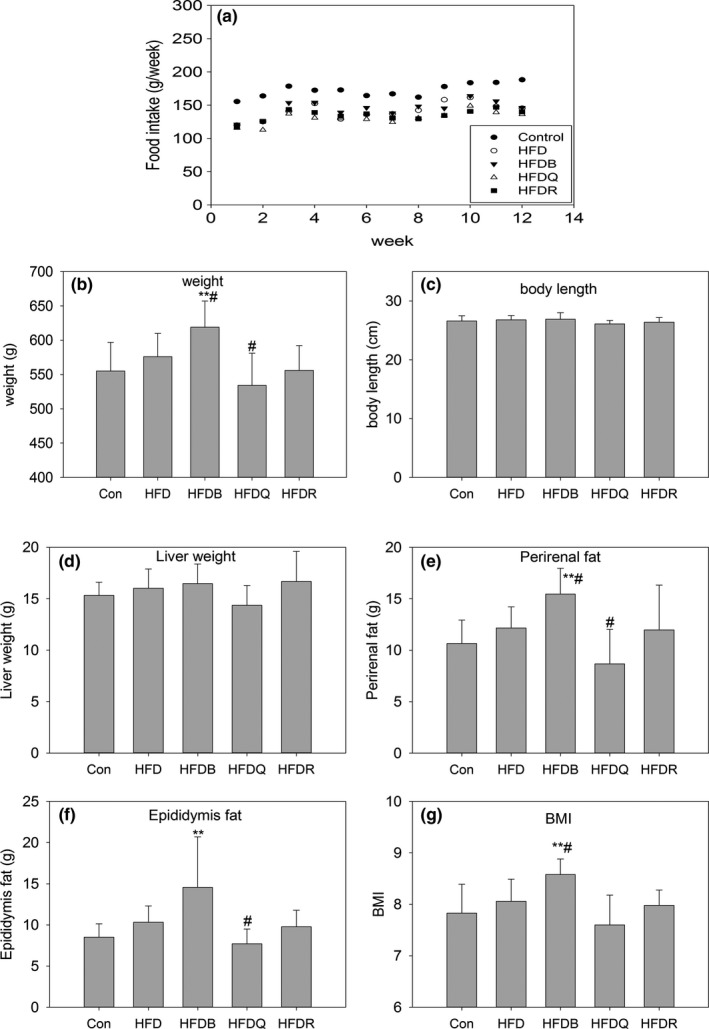
Effect of tartary buckwheat and flavonoids on food intake (a), body weight (b), body length (c), liver weight (d), perirenal fat (e), epididymis fat (f), and body mass index (g). ^**^ *p* < .01 and ^*^ *p* < .05 denote statistically significant differences with control group; ^##^ *p* < .01 and ^#^ *p* < .05 denote statistically significant differences with HFD group

**Figure 2:**
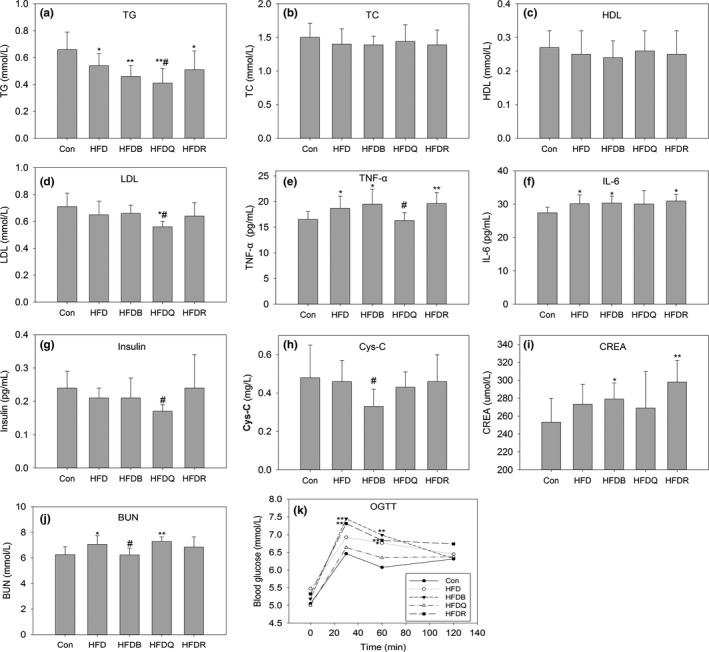
Effect of Tartary buckwheat and flavonoids on serum triacylglycerol (a), total cholesterol (b), high‐density lipoprotein cholesterol (c), low‐density lipoprotein cholesterol (d), TNF‐α (e), IL‐6 (f), insulin (g), cystatin C (h), creatinine (i), urea (j), and oral glucose tolerance test (k). ^**^ *p* < .01 and ^*^ *p* < .05 denote statistically significant differences with control group; ^##^ *p* < .01 and ^#^ *p* < 0 0.05 denote statistically significant differences with HFD group

In order to evaluate the effect of Tartary buckwheat on lipid metabolism in rats during high‐fat diet, 0.2% rutin and 0.1% quercetin were used as reference (calculated based on the assumption that daily intake of 10% Tartary buckwheat (contained 2% rutin) of total intake, and at this point, animal feed contained about 0.2% rutin or 0.1% quercetin, which was degraded from rutin by rutin degraded enzyme during processing). The information about the composition of Tartary buckwheat (passed through a 80–120 mesh) used in the diet of HFDB was as follows: 2.74% rutin, 0.08% quercetin, 53.4% starch, 1.74% fiber, 16.4% protein, and 4.2% fat. Figures 1 and 2 show that after 12 weeks of high‐fat feeding, the body weight, and serum TC and TG of HFD rats did not increase compared with the control group. In contrast, TC values of HFD, HFDQ, HFDR, and HFDB groups were lower than that in the control group, agreeing with Lundsgaard *et al*., where HFD feeding lowered the TG content in mice and humans (Lundsgaard et al., 2019). HDL and LDL did not change significantly. In the present study, quercetin decreased food intake and significantly reduced body weight, perirenal fat, epididymis fat, serum TG, and LDL compared with HFD, but rutin showed little effect on this basis although there was a trend for TG to decrease, and this effect was not statistically significant. A previous report has indicated that rutin significantly decreased TC, TG, HDL, and LDL (Hsu, Wu, Huang, & Yen, 2009). Another report showed that rutin affected TG, but had little effect on TC, HDL, and LDL (Zhang et al., 2017). Previous reports have indicated that 22 days of continuous ingestion of rutin in a dose‐dependent manner (from 10 to 1,000 mg/kg) in rats affected neither the level of lipid metabolism parameters in the serum and liver nor the level of steroid excretion into feces (Nakamura, Ishimitsu, & Tonogai, 2008). These inconsistencies may be explained by the different diets, doses, and animal models used in these studies.

It was surprising that Tartary buckwheat bran significantly increased the weight of rats, perirenal fat, and epididymis fat content and increased food intake compared with HFD although HFDB was rich in rutin (0.27%). Another study reported no significant differences in the atherosclerosis index andoxidized LDL levels, and in lipid metabolism parameters between the Tartary buckwheat food and the placebo food groups (Nishimura et al., 2016). The rutin content of Tartary buckwheat is estimated to be approximately 100 times that of common buckwheat (Yasuda & Nakagawa, 1994), but no differences were reported between the two types of buckwheat on the serum total cholesterol levels in another study (Wieslander et al., 2011). Thus, it might be deduced that there were effects caused by components in the buckwheat other than rutin. Our previous report has shown that Tartary buckwheat bran was rich in rutin, and contained much higher levels of fatty acids, and amino acids than Tartary flour (Peng et al., 2017). In the present study, the buckwheat and wheat flour were steamed to make them more similar to a human diet. Looking at these two studies, we can suggest that the nutrients available in Tartary buckwheat were connected to its components and processing method and that more attention should be paid to these differences.

The TNF‐α and IL‐6 contents increased significantly in the HFD, HFDR, and HFDB groups, but quercetin significantly reduced their contents relative to HFD, implying that quercetin may reduce fat accumulation by improving the state of inflammation in body. The high‐fat di*et al*so increased serum urea and creatinine levels, thus increasing the risk of overburdening the kidneys. It was interesting to note that buckwheat decreased the urea and creatinine levels, suggesting that buckwheat has the potential to protect renal function. Twelve weeks of quercetin dietary treatment improved glucose tolerance as shown by the reduced blood glucose levels in the oral glucose tolerance test (OGTT), but the rutin and Tartary buckwheat treatments had no similar effects. These findings suggested that quercetin administration could improve glucose tolerance and insulin resistance, and alleviate fat accumulation in diet‐induced obese rats.

**Figure 3:**
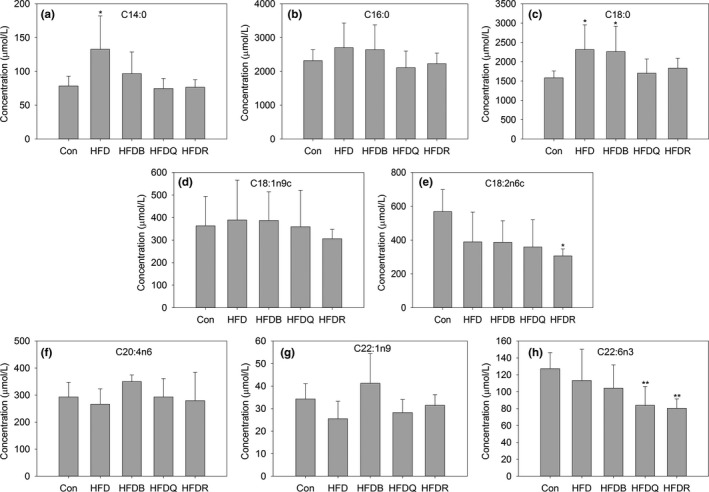
Effect of Tartary buckwheat and flavonoids on serum fatty acid. ^**^ *p* < .01 and ^*^ *p* < .05 denote statistically significant differences with control group; ^##^ *p* < .01 and ^#^ *p* < .05 denote statistically significant differences with HFD group

Regarding fatty acids (FAs), eight types of FA were detected in the serum of rats by gas chromatography at the 12th week for the control and HFD groups. Figure 3 shows that the concentrations of C16:0, C18:0, C18:1, C18:2, and C20:4 in rat serum were much higher than those of the other FAs, findings consistent with other reports (Wang et al., 2016; Zhukova, Novgorodtseva, & Denisenko, 2014). Saturated fatty acids, such as C14:0, C16:0, and C18:0, which can promote inflammation in the body, were higher in the HFD group. Administering flavonoids tended to reduce the contents of these FAs.

### 3.2 Histopathology

**Figure 4:**
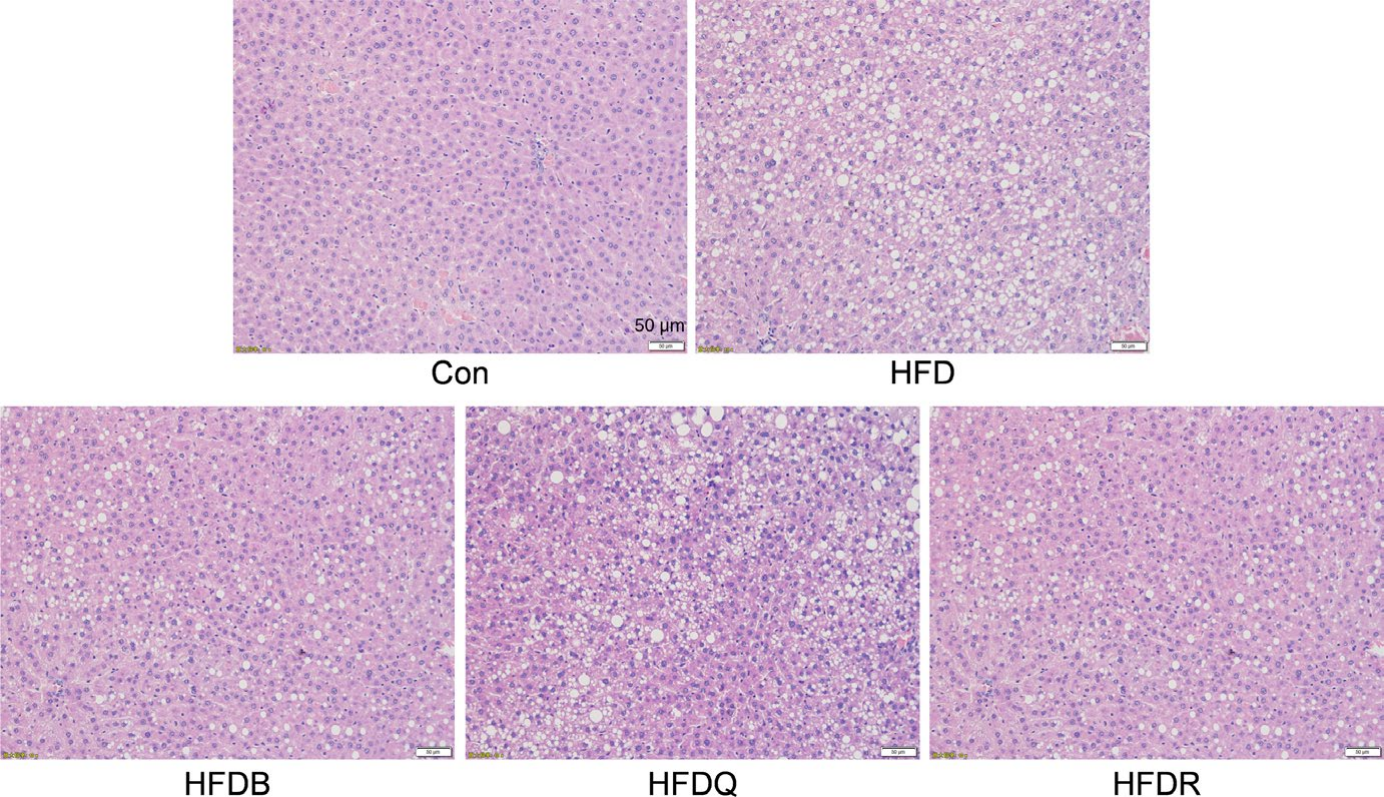
Effect of Tartary buckwheat and flavonoids on liver histology and lipid accumulation in HFD‐fed rat, using Hematoxylin & Eosin staining method

Flavonoids have been found to have positive effects on lipid metabolism, insulin resistance, inflammation, and oxidative stress, the most important pathological processes in the etiology of hepatic steatosis even to nonalcoholic fatty liver disease (NAFLD)(Van De Wier, Koek, Bast, & Haenen, 2017). The development of steatohepatitis in animals on a high‐fat diet not only depends on the rodent species and strain but also on the fat content in the diet, the composition of dietary fat, and the duration of treatment (Takahashi, Soejima, & Fukusato, 2012). NAFLD is a multi‐factorial disease, so it is difficult to imitate all the facets of the disease in one animal model. The models using rats or gerbils on a high‐fat diet or on a high‐fat diet combined with high fructose or carbohydrates seem to best approximate to the human conditions of NAFLD (Van De Wier et al., 2017). Figure 4 shows that although a high fat intake lowered serum lipids, the histopathological assessment of the livers of mice fed HFD revealed microvesicular and macrovesicular steatosis in hepatocytes, compared with the control mice, thus indicating a disturbed lipid metabolism. One review has indicated that the administration of flavonoids such as quercetin or rutin led to a decrease in steatosis (Van De Wier et al., 2017). A recent report has found that quercetin supplementation reduced intrahepatic lipid accumulation significantly through its ability to modulate lipid metabolism gene expression, cytochrome P450 2E1 (CYP2E1)‐dependent lipoperoxidation, and related lipotoxicity (Porras et al., 2016). However, in the present study, these flavonoids and Tartary buckwheat only exhibited a weak effect on fat deposition. This may be explained by the different diets, doses, animal models, and treatment times, and therefore, further systematic studies are required to take account of these complicating factors.

### 3.3 SCFAs production

**Figure 5:**
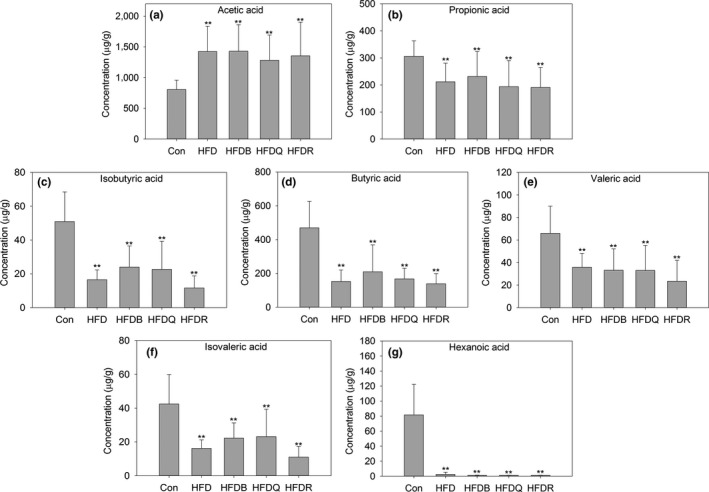
Effects of Tartary buckwheat and flavonoids on fecal short‐chain fatty acid production. ^**^ *p* < .01 and ^*^ *p* < .05 denote statistically significant differences with control group; ^##^ *p* < .01 and ^#^ *p* < .05 denote statistically significant differences with HFD group

SCFAs, such as acetate, propionate, and butyrate, are produced by the intestinal microbiota and are perhaps the most extensively studied molecules, which mainly influence the host's energy metabolism and appetite (Wong, Souza, Kendall, Emam, & Jenkins, 2006). In the present study, isobutyric, isovaleric, pentanoic, and hexanoic FA were also quantified in the cecal samples by our GC‐MS method, which provided a good separation and suitable retention time for SCFAs. Previous studies have disagreed on how alterations in SCFAs affect the development of obesity. Many studies have reported that overweight and obese individuals have increased concentrations of SCFAs (Schwiertz et al., 2010; Turnbaugh et al., 2006). However, SCFA supplementation tends to reduce body weight in both humans and rodents (Chambers et al., 2015; Lu et al., 2016). In mice, acetate in the colon has been shown to induce the secretion of glucagon‐like peptide (GLP)‐1, which reduces food intake and obesity (Tolhurst et al., 2012). Another study has proposed that circulating acetate enhances insulin secretion through a microbiome‐brain‐β‐cell axis, and promotes obesity and the metabolic syndrome (Perry et al., 2016). Propionate is primarily used by hepatocytes, so gut‐derived propionate has been shown to reduce liver fat in adults with NAFLD (Brass & Beyerinck, 1988). Previous studies have indicated that dietary propionate and butyrate supplementation ameliorates body weight gain, oral glucose tolerance, TG concentrations, and fasting insulin in mice (Lin et al., 2012; Lu et al., 2016). In the present study, Figure 5 shows that high‐fat diet rats exhibited significantly higher levels of acetate (1,424.63 μg/g) but lower levels of propionate (211.66 μg/g), butyrate (152.84 μg/g), and other SCFAs than the control group. These results agreed with the study of Perry et al. (2016), but were inconsistent with that of Porras (Porras et al., 2016) where HFD decreased the acetate, propionate, and butyrate contents. Differences in doses, patterns, species, timing, and composition of feedstuff are plausible explanations for these discrepancies. Thus, more research is required to resolve these conflicts regarding the role of SCFAs in the metabolic syndrome. Rutin, quercetin, or Tartary buckwheat dietary treatment had little effect on the production of SCFAs, although the acetate content tended to decrease for the quercetin diet (1,424.63 to 1,281.18 μg/g), but the effect was not statistically significant. Propionate and butyrate contents decreased dramatically in the HFD, HFDQ, HFDR, and HFDB groups (306.11 to 211.66, 193.87, 190.99, and 231.39 μg/g, respectively and 470.04 to 152.84, 167.36, 138.61, and 209.74 μg/g, respectively). These effects may have been mediated by the gut microbiota and increased steatosis in the liver and serum inflammation.

### 3.4 Gut microbiota

**Figure 6:**
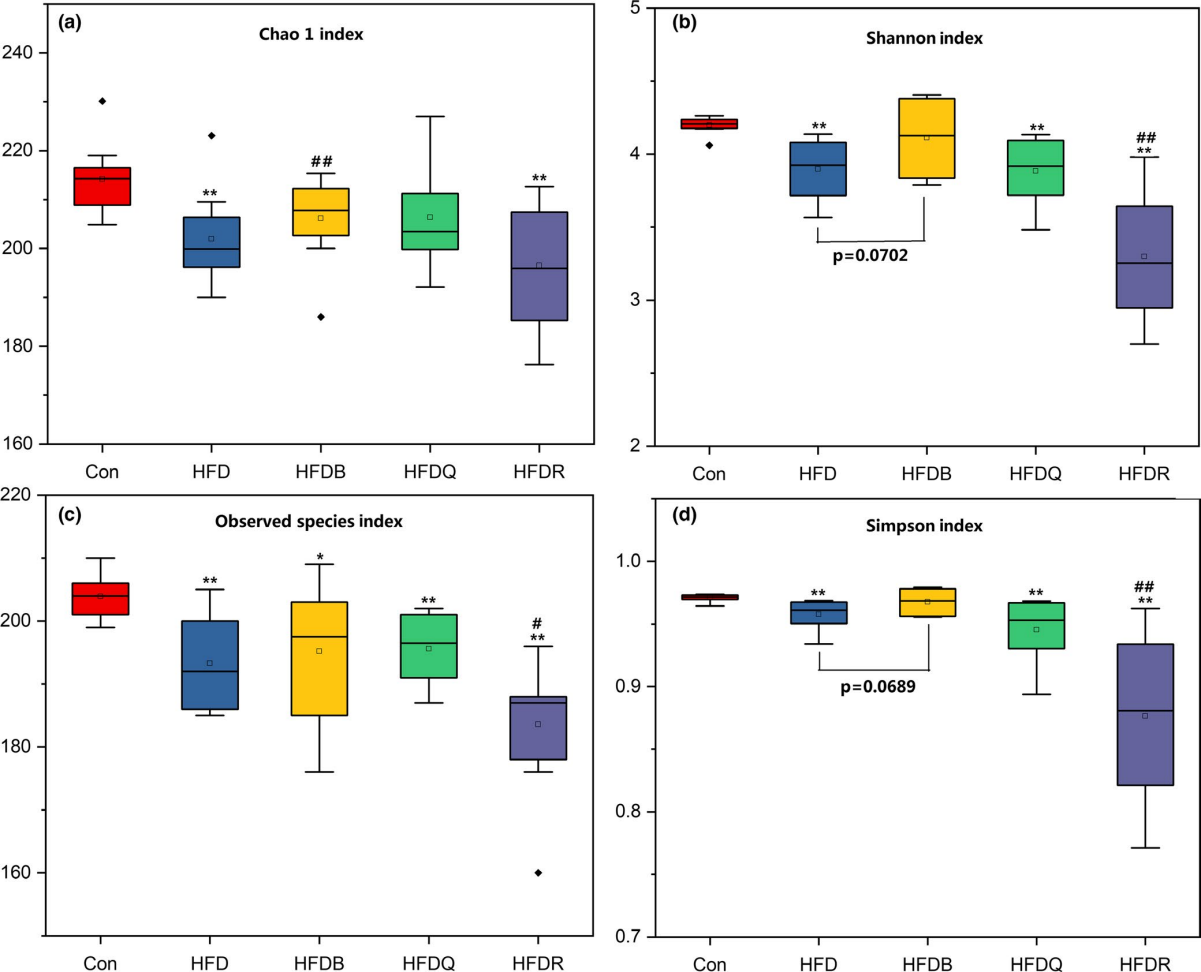
Effects of Tartary buckwheat and flavonoids on the alpha diversity of gut microbiota. ^**^ *p* < .01 and ^*^ *p* < .05 denote statistically significant differences with control group; ^##^ *p* < 0 0.01 and ^#^ *p* < .05 denote statistically significant differences with HFD group

To assess the effects of flavonoids and buckwheat treatments on the microbial composition of the gut microbiome, the V3‐V4 hypervariable region of the 16S rDNA gene was sequenced. A total of 1,840,616 reads were obtained from the cecal samples. After denoising and filtering, 1,707,033 clean tags were used for subsequent analysis. Based on a 97% similarity level, all of the effective reads were clustered into OTU. Compared with the control group, the HFD treatment significantly reduced the number of OTUs. Figure 6 shows that other microbial diversity indices, the Shannon index, Chao1 index, Simpson index, and Observed species index decreased in the HFD group. Tartary buckwheat treatment increased Chao1 index significantly, and this may be an important potential beneficial effect for human. Quercetin tends to increased Chao1 index, but not reach significant.

**Figure 7:**
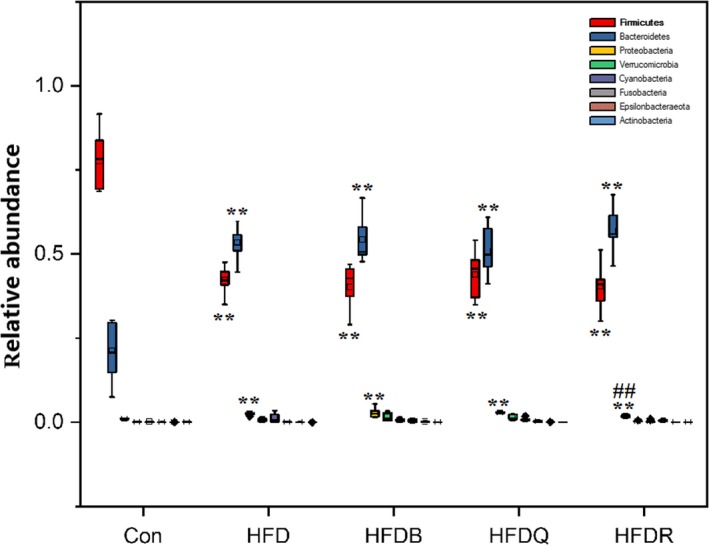
Effects of Tartary buckwheat and flavonoids on gut microbiota balance in HFD‐fed rats (phylum level). ^**^ *p* < .01 and ^*^ *p* < .05 denote statistically significant differences with control group; ^##^ *p* < .01 and ^#^ *p* < .05 denote statistically significant differences with HFD group

Most of the identified reads from the cecal content of mice were classified within three phyla. The *Firmicutes* phylum was predominant, representing 49% of the 16S rDNA gene sequences, followed by *Bacteroidetes* (47%) and *Proteobacteria* (2%). These three phyla represented more than 95% of the sequences analyzed. Figure 7 shows the relative bacterial composition at the phylum level for each group. The relative abundance of the *Bacteroidetes* and *Proteobacteria* phyla was significantly higher when *Firmicutes* was lower in the HFD groups compared with the control groups. These results were inconsistent with those of Porras *et al* (Porras et al., 2016). The review by Tagliabue and Elli discussed how obesity in humans has been associated with reduced bacterial diversity and an altered representation of bacterial species, but the identified differences were not consistent among the studies (Tagliabue & Elli, 2013). They concluded that the question still remains as to whether changes in the intestinal microbial community are one of the environmental causes of overweight and obesity or whether they are a consequence of obesity, specifically of the unbalanced diet that often accompanies the development of excess weight gain. These studies combined with the findings of the present study suggest that the relationship between bacterial composition at the phylum level and rats fed a high‐fat diet need further clarification and that more confounding variables such as diet composition should be take into account. The diets in the HFDQ, HFDR, and HFDB groups showed little effect on changing these three phyla (*Firmicutes*, *Bacteroidetes,* and *Proteobacteria*).

**Figure 8:**
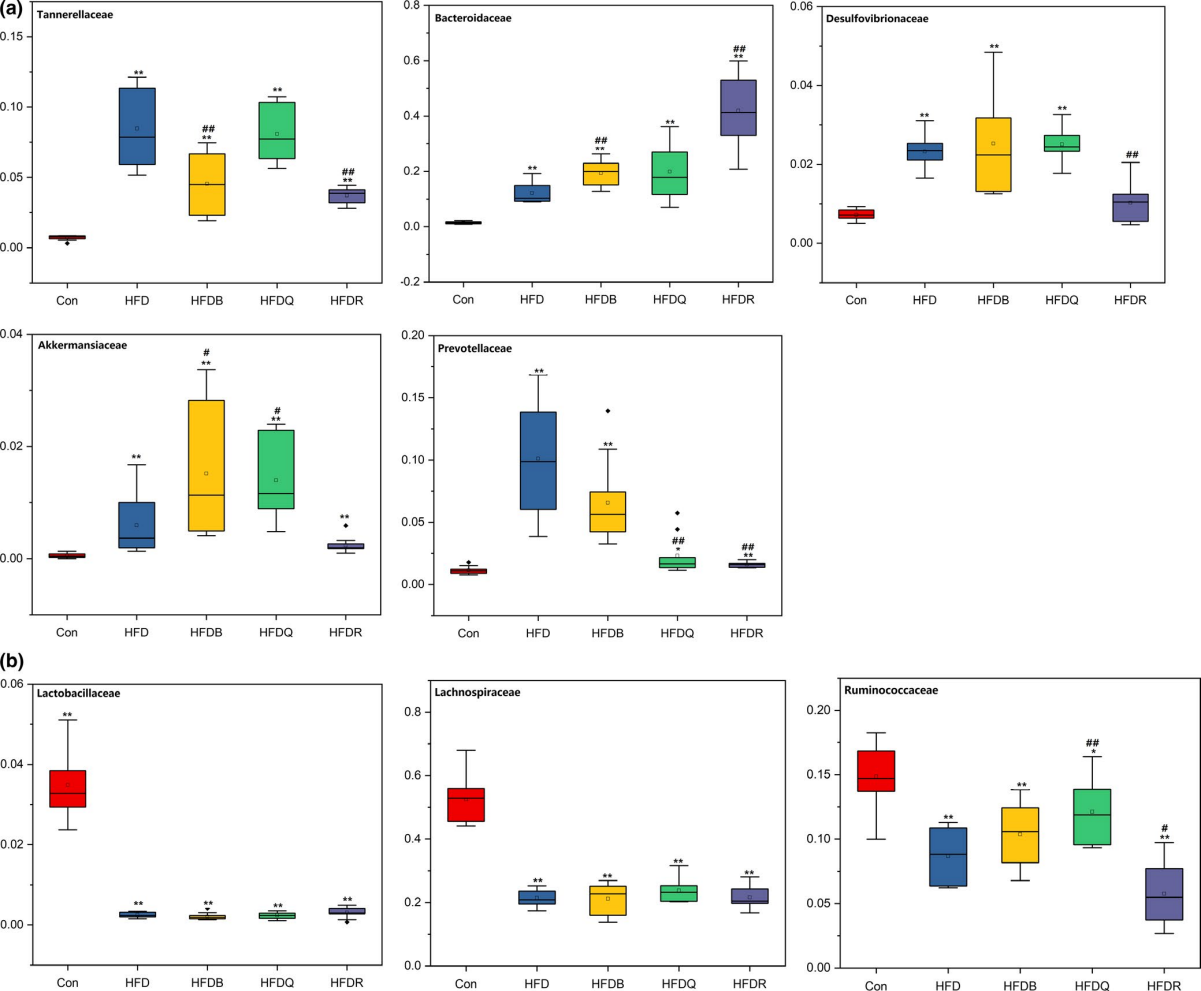
Effects of Tartary buckwheat and flavonoids on gut microbiota balance in HFD‐fed rats (family level). ^**^ *p* < .01 and ^*^ *p* < .05 denote statistically significant differences with control group; ^##^ *p* < .01 and ^#^ *p* < .05 denote statistically significant differences with HFD group

Figure 8 shows the effects of Tartary buckwheat and flavonoids on the balance of gut microbiota at the family level in HFD‐fed rats. *Lachnospiraceae*, which may protect against colon cancer in humans by producing butyric acid (Meehan & Beiko, 2014), significantly decreased in the HFD, HFDQ, HFDR, and HFDB groups. These results were consistent with the changes in butyric acid content. *Lachnospiraceae* has been found to cause diabetes in germ‐free mice (Kameyama & Itoh, 2014). *Pr* *evotellaceae*, composed of four genera, which can be opportunistic pathogens, increased significantly in the HFD group but decreased in the HFDB, HFDQ, and HFDR groups. Consistent with results at the phylum level, the abundance of *Bacteroidaceae* increased significantly in the HFD, HFDB, and HFDQ groups. *Bacteroidaceae* increased significantly in the HFDR group compared with the HFD group. The abundance of *Tannerellaceae*, *Desulfovibrionaceae,* and *Akkermansiaceae* was much higher in the HFD group, but was significantly reduced by rutin treatment, but quercetin had no effect. Quercetin also increased the abundance of *Ruminococcaceae* to approach that of the control: These may be the important differences in the effect of rutin and quercetin. Figure 8 also shows the changes in the main microbiota at the family level.

**Figure 9:**
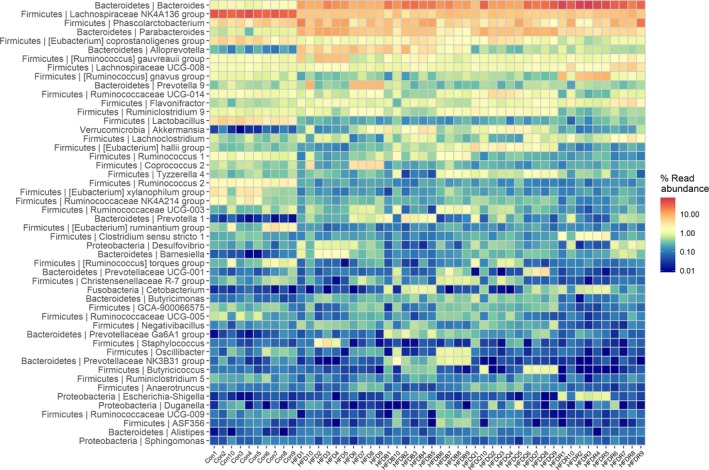
Heat map of gut microbiota (genus level)

**Figure 10:**
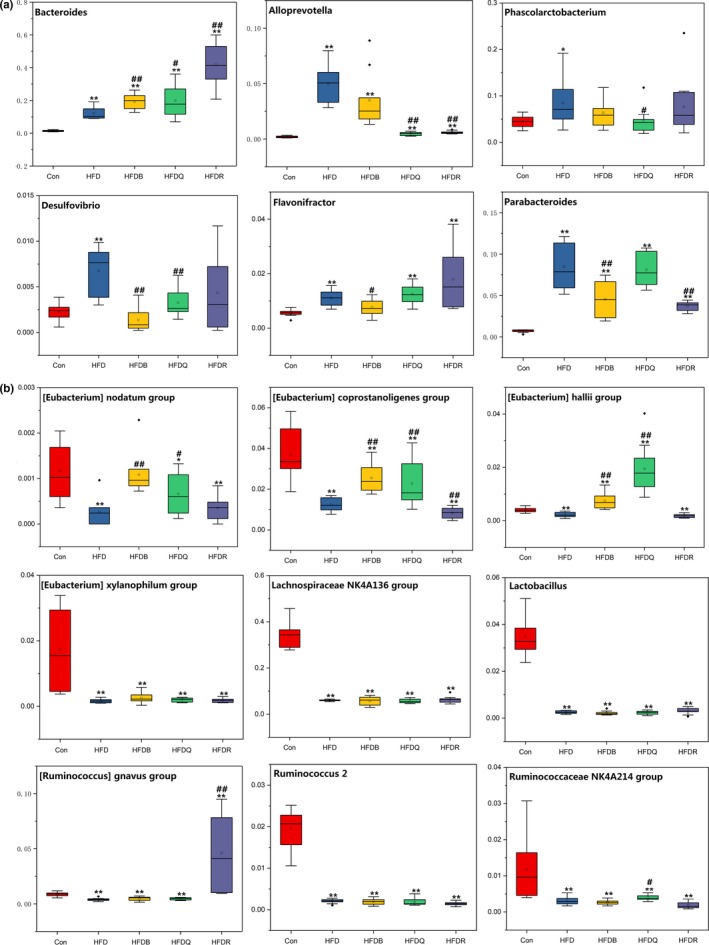
Effects of Tartary buckwheat and flavonoids on gut microbiota balance in HFD‐fed rats (genus level). ^**^ *p* < .01 and ^*^ *p* < .05 denote statistically significant differences with control group; ^##^ *p* < .01 and ^#^ *p* < .05 denote statistically significant differences with HFD group

At the genus level, most of the sequences belonged to the genera, *Bacteroides*, *Lachnospriraceae* NK4A136 group, *Phascolarctobacterium*, *Parabacteroides,* and *Ruminococcus*. Polyphenols may decrease carbohydrate absorption, thus affecting the supply of carbohydrates required for microbial growth in the GI tract, particularly saccharolytic bacteria such as *Bacteroides*, *Bifidobacterium*, *Clostridium*, *Eubacterium*, *Lactobacillus*, and *Ruminococcus *(Maukonen & Saarela, 2015). Figures 9 and 10 were consistent with the results at the family level, showing that the abundance of *Bacteroides* was higher in the HFD group than in the control group. Treatment with buckwheat and flavonoids further increased the *Bacteroides* level especially in the HFDR group. *Bacteroides* have been suggested as a fecal indicator organism because they make up a significant portion of the fecal bacterial population, and have a high degree of host specificity that reflects differences in the digestive system of the host animal (Bernhard & Field, 2000). However, their effected on lipid metabolism needs further clarification. The *Lachnospiraceae*NK4A136 group belongs to the *Lachnospiraceae* family and, in our study, was significantly decreased by a high‐fat diet. *Alloprevotella*, which can produce acetic acid, increased significantly in the HFD group, but were restored to similar levels as the control group in the HFDQ and HFDR groups. However, Tartary buckwheat had little effect on *Alloprevotella*, although it contained a high level of rutin. *Desulfovibrio* and *Phascolarctobacterium* were higher in the HFD group, but quercetin restored their abundance to similar levels as the control group. *Eubacterium*, the mainly genus converting flavonoids, was much lower in the HFD, but Tartary buckwheat and quercetin significantly increased the abundance of this genus. Treatment with Tartary buckwheat and flavonoids did not increase the relative abundance of *Lactobacillus*, which suggested a positive link with weight control. The main change in the other genera is shown in Figures 9 and 10.

**Figure 11:**
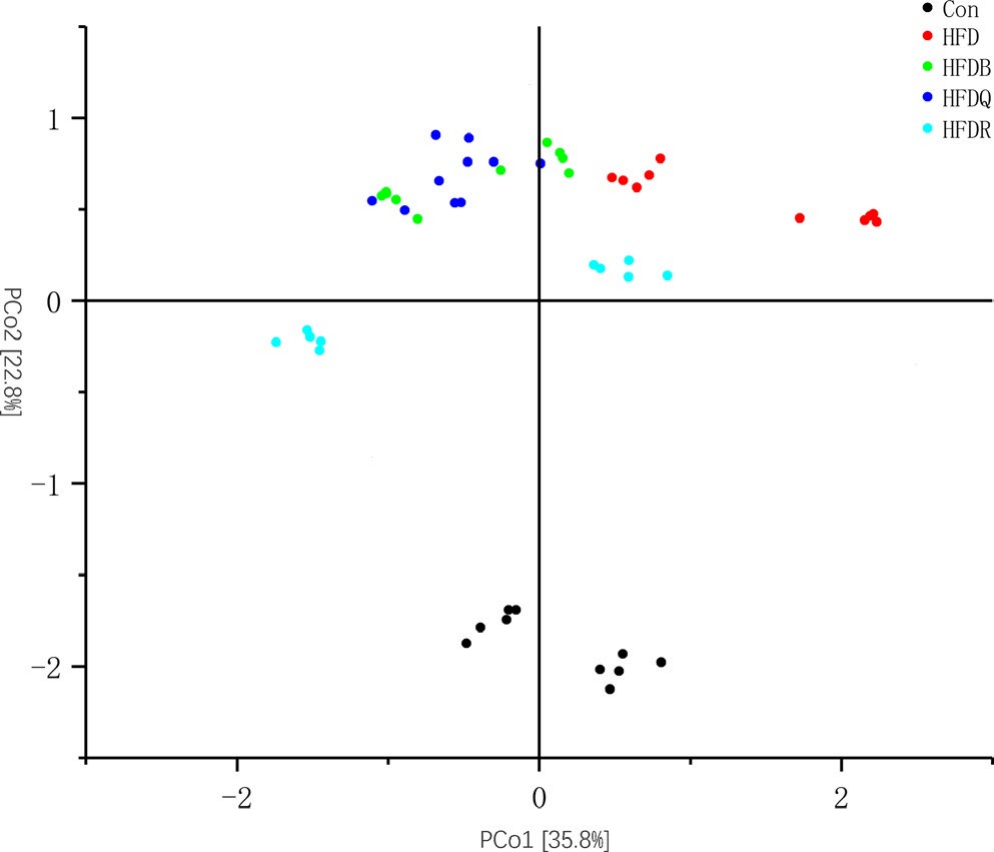
Principal coordinates analysis (PCoA) plot based on Bray–Curtis distance at the genus level between the five groups

Beta diversity measurements were performed on the cecal content to determine whether there were any differences in global bacterial composition between the five treatment groups. A PCoA based on the Bray–Curtis distance was performed (Figure 11) showing that the bacterial communities of rats fed with HFD clustered together according to the second axis (22.8%) based on the diet. For the HFD rats with or without treatment with Tartary buckwheat or flavonoids, the communities of the HFDR group were separate from the others on the second axis (22.8%). The HFD group could also be distinguished from the HFDB and HFDQ groups on the first axis (35.8%). However, the HFDQ and HFDB groups were dispersed along these two axes, although the weight of rats treated with Tartary buckwheat and quercetin was significantly different. This result implied that the relationship between weight gain and gut microbiota was complex, and more evidence was needed. One interesting finding was the similarity in the microbiota composition of rats from the same cage, with some differences from rats in other cages from the same treatment group. This also implied that the community of rats has an important effect on microbiota structure and may need to be taken into consideration in any further research.

In conclusion, our findings suggest that quercetin could attenuate HFD‐induced obesity and related metabolic disorders in rats. This may have been caused by its antioxidant activity and inhibitory effect on inflammation. Rutin had little effect on attenuating obesity, but tended to decrease fat deposition in the liver. It was surprising that Tartary buckwheat increased body weight and fat accumulation, despite showing the effect of attenuating hepatic steatosis. HFD increased the fecal acetate content but decreased other SCFAs. Tartary buckwheat, quercetin, and rutin had little effect on the production of SCFAs. Tartary buckwheat, quercetin, and rutin treatments all modulated the gut microbiota, but more research is needed to clarify the relationship between gut microbiota and obesity. Meanwhile, the role of quercetin and rutin in the diet needs more systematic assessment so that confounding variables can be taken into account and techniques can be standardized.

## CONFLICT OF INTEREST

The authors declare that they do not have any conflict of interest.

## ETHICAL APPROVAL

All animals used in this study were reared and euthanized with the approval of the Use Committee of Chengdu University. All experiments were performed following the guidelines and regulations of “the instructive notions with respect to caring for laboratory animals” issued by the Ministry of Science and Technology of the People's Republic of China.
