# Correction to “Effect of tartary buckwheat, rutin, and quercetin on lipid metabolism in rats during high dietary fat intake”

**DOI:** 10.1002/fsn3.70207

**Published:** 2025-04-25

1

Peng L, Zhang Q, Zhang Y, et al. Effect of tartary buckwheat, rutin, and quercetin on lipid metabolism in rats during high dietary fat intake. *Food Sci Nutr*. 2020; 8: 199–213. https://doi.org/10.1002/fsn3.1291

The Correspondence section contained errors. The correct information is as follows:

**Correspondence**

Zhu‐Yun Yan, Pharmacy College, Chengdu University of Traditional Chinese Medicine, Chengdu 611137, China.

Email: yanzhuyun@cdutcm.edu.cn

Gang Zhao, Key Laboratory of Coarse Cereal Processing of Ministry of Agriculture and Rural Affairs, Chengdu University, Chengdu 610106, China.

Email: zhaogang@cdu.edu.cn

In Figure 4, the image for Group HFDB was mistakenly duplicated in Group HFDR. This mistake occurred during the process of assembling the figure and does not affect the results, analysis, or conclusions presented in the manuscript.

**FIGURE 4:**
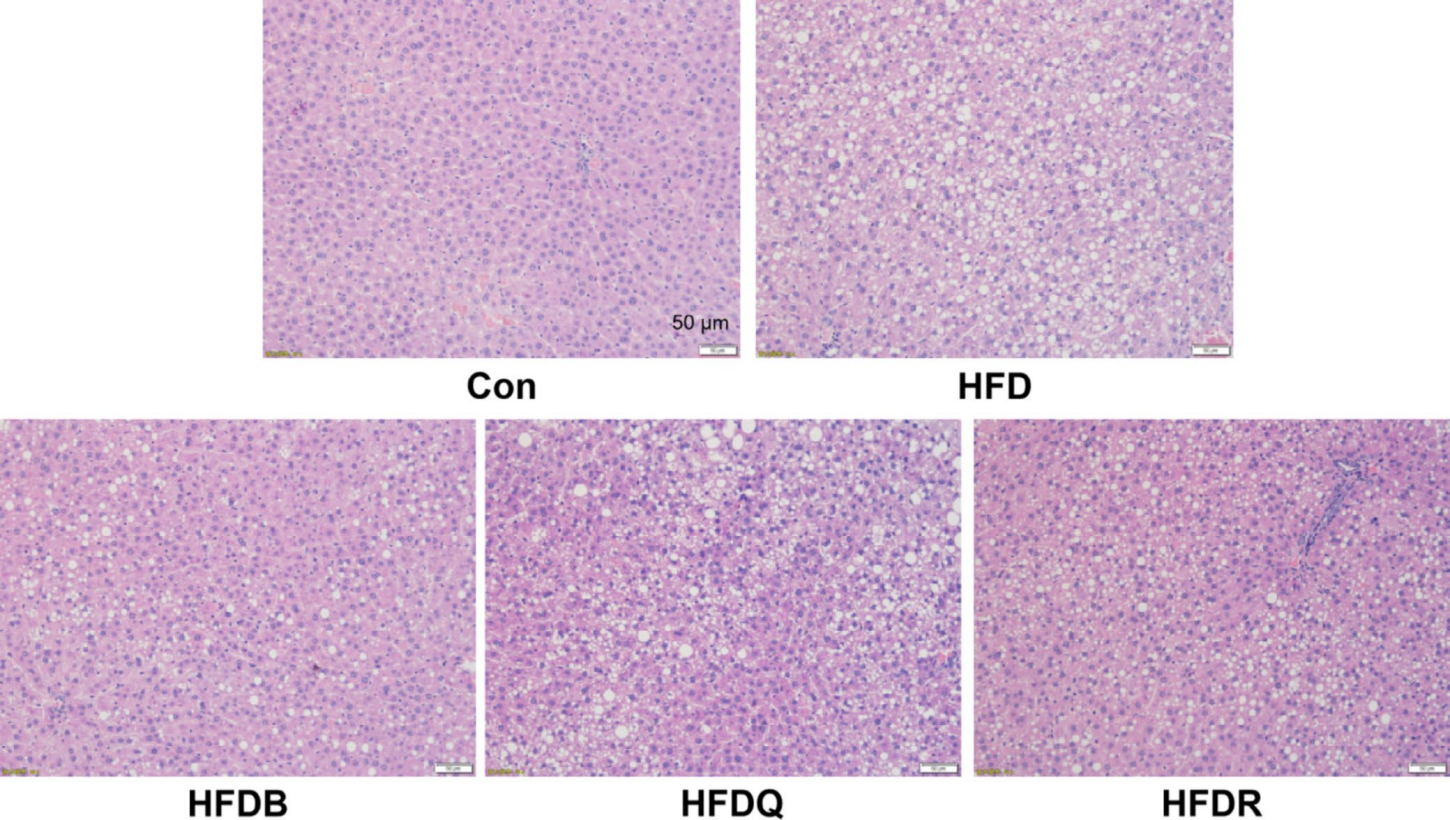
Effect of Tartary buckwheat and flavonoids on liver histology and lipid accumulation in HFD‐fed rat, using Hematoxylin & Eosin staining method.

The corrected Figure 4 appears below.

We apologize for this error.

